# TSC2/PKD1 contiguous deletion syndrome in a pregnant woman: A case report

**DOI:** 10.3389/fmed.2023.1101079

**Published:** 2023-02-21

**Authors:** Shaofang Huang, Kangxiang Xu, Yuqi Xu, Lu Zhao, Xiaoju He

**Affiliations:** ^1^Department of Obstetrics and Gynecology, The Second Affiliated Hospital of Nanchang University, Nanchang, China; ^2^Second Clinical Medical College, Nanchang University, Nanchang, China

**Keywords:** TSC2/PKD1 contiguous gene deletions, pregnant woman, renal angiomyolipoma, tuberous sclerosis, polycystic kidney disease, prenatal diagnosis

## Abstract

TSC2/PKD1 contiguous gene deletion syndrome is a disease caused by the deletions of the *TSC2* and *PKD1* genes. This is a rare contiguous genomic disease with clinical manifestations of tuberous sclerosis and polycystic kidney disease. To our knowledge, this case report is the first known case of TSC2/PKD1 contiguous gene deletions in a pregnant woman. The patient had multiple renal cysts, angiomyolipoma, hypomelanotic macules, shagreen patch, subependymal giant cell astrocytoma, multiple cortical tubers, and subependymal nodules. The patient underwent genetic testing. To exclude genetic defects in the fetus, prenatal fetal genetic testing was performed after obtaining the patient’s consent. We found an increasing trend in the size of renal cysts and renal angiomyolipomas in patients with polycystic kidney with tuberous sclerosis during pregnancy. Through enhanced clinical monitoring of patients and prenatal genetic testing of the fetus, timely and effective clinical intervention for the mother may be achieved, thus obtaining the best possible outcome for both mother and fetus.

## 1. Introduction

The TSC2/PKD1 contiguous gene deletion syndrome (MIM# 600273), also known as polycystic kidney disease with tuberous sclerosis (PKDTS), is a contiguous gene deletion syndrome involving the chromosome 16p13.3 on *PKD1* and *TSC2* genes. PKDTS was first reported by Brook-Carter et al. (1). Mutations in the *TSC2* gene cause approximately 69% of all tuberous sclerosis complex (TSC) cases (2). TSC is a multisystemic genetic disorder with an incidence of 1 per 6,000–10,000 live births (3). Its clinical presentation is diverse and varies from person to person, with the main symptoms being malformations of multiple organ systems including the brain, skin, heart, kidneys, and lungs. Complications of TSC include epilepsy, learning difficulties, behavioral problems, and renal failure. Almost all patients with TSC have skin lesions such as depigmentation, facial angiofibromas, shagreen patch, brown fibrous plaques, and nail fibromas (4). The main clinical features of autosomal dominant polycystic kidney disease (ADPKD) are renal cysts, hepatic cysts, intracranial aneurysms, and heart valve lesions. A common complication of ADPKD is kidney stones, with end-stage renal disease being its most serious complication and the leading cause of mortality (5). The most common renal lesions are renal cysts and vascular smooth muscle lipomas in patients with TSC. A review of the relevant literature reveals that PKDTS is broadly an overlay of symptoms of both TSC and polycystic kidney disease (PKD), in addition to the clinical renal manifestations. PKDTS exhibits more severe polycystic kidney growth with early renal impairment and early end-stage renal failure than TSC and ADPKD (6). To the best of our knowledge, there are no definitive clinical guidelines for the diagnosis and treatment of PKDTS to date.

## 2. Case report

A 21-year-old pregnant woman was followed-up by an obstetrician and gynecologist for bilateral cystic nephropathy and angiomyolipoma since the 13th week of pregnancy. Upon admission, the patient’s urine protein was negative and renal function was poor [serum uric acid (UA), 572.74 μmol/L; serum creatinine (Scr), 302.98 μmol/L; urea, 22.48 mmol/L]. At 16 weeks, the urine protein was still negative but serum UA had increased (598.91 μmol/L); Scr (291.62 μmol/L); and urea (15.23 mmol/L) levels had decreased. At 23 weeks, the patient’s Scr was increased (332.15 μmol/L), the UA was 594.80 μmol/L, and urea was 18.29 mmol/L. At 30 weeks, the patient had an increase in UA, Scr, and urea (UA, 644.39 μmol/L; Scr, 343.46 μmol/L; and urea, 29.8 mmol/L). The characteristics of both TSC and PKD suggest dual pathogenesis; thus, we clinically suspected PKDTS. During the patient’s hospitalization, we carried out multi-disciplinary treatment and informed the patient of the possible risks during pregnancy, including complications such as end-stage renal failure, ruptured renal angiomyolipoma (RAML), and bleeding. The patient was also informed of the possible need for renal replacement therapy as the disease progressed and the possible need for super-selective intra-arterial embolization for RAML. We recommended termination of the pregnancy, but the patient refused termination and opted for conservative treatment. We conducted molecular analysis to delineate the underlying genetic etiology.

## 3. Physical examinations and findings

**FIGURE 1:**
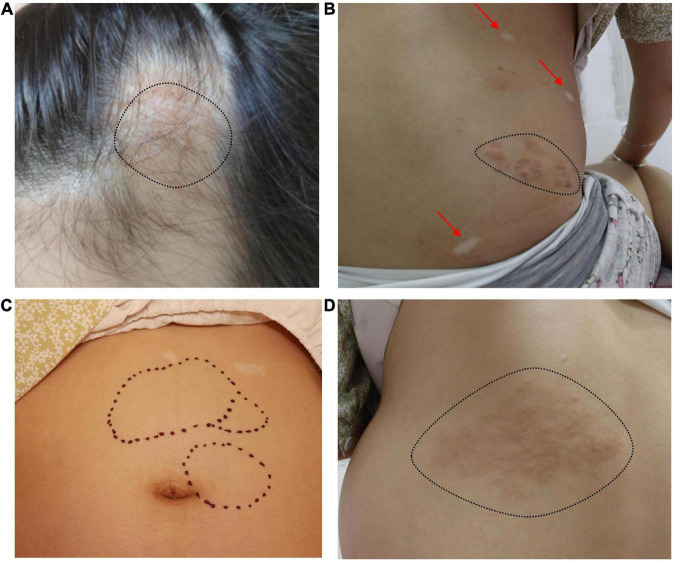
**(A)** A soft tissue mass can be seen on the left cranial top near the forehead (black dotted line). **(B)** Hypomelanotic macules in multiple locations throughout the body (red arrow). Shagreen patch (black dotted line). **(C)** Multiple masses can be palpated on the left side of the abdomen (black dotted line) and hypomelanotic macules can be seen in the upper abdomen. **(D)** Shagreen patch (black dotted line).

A soft tissue mass could be seen on the cranial top near the forehead (Figure 1A). Hypomelanotic macules were found in multiple locations throughout the body (Figures 1B, C). Multiple masses could be palpated on the left side of the abdomen (Figure 1C), and a shagreen patch was seen on the face and lower back (Figures 1B, D).

## 4. Ultrasonography findings

**FIGURE 2:**
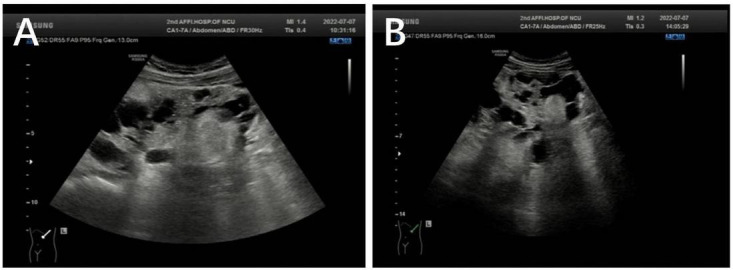
**(A)** Left polycystic renal lesions (maximum cystic diameter: 25 × 24 mm) and multiple solid masses (maximum solid diameter: 54 × 38 mm) were seen on ultrasonography at the 13th week of pregnancy. **(B)** Left polycystic renal lesions (maximum cystic diameter: 55 × 35 mm) and multiple solid masses (maximum solid diameter: 75 × 60 mm) were seen on ultrasonography at the 24th week of pregnancy.

Bilateral polycystic renal lesions (maximum cystic diameter: right 30 × 27 mm, left 25 × 24 mm) and multiple solid masses (maximum solid diameter: right 26 × 25 mm, left 54 × 38 mm) were seen on ultrasonography during the 13th week of pregnancy. We conducted a follow-up observation for about 3 months from the patient’s 13th week of pregnancy, in which MRI showed a trend of increasing size of renal cysts and RAML s. Bilateral polycystic renal lesions (maximum cystic diameter: right 42 × 32 mm, left 55 × 35 mm) and multiple solid masses (maximum solid diameter: right 41 × 29 mm, left 75 × 60 mm) were seen on ultrasonography at the 24th week of pregnancy. Bilateral polycystic renal lesions (maximum cystic diameter: right 42 × 32 mm, left 55 × 35 mm) and multiple solid masses (maximum solid diameter: right 46 × 32 mm, left 59 × 47 mm) were seen on ultrasonography at the 30th week of pregnancy. The multiple solid masses were thought to be RAML (Figure 2).

## 5. MRI findings

**FIGURE 3:**
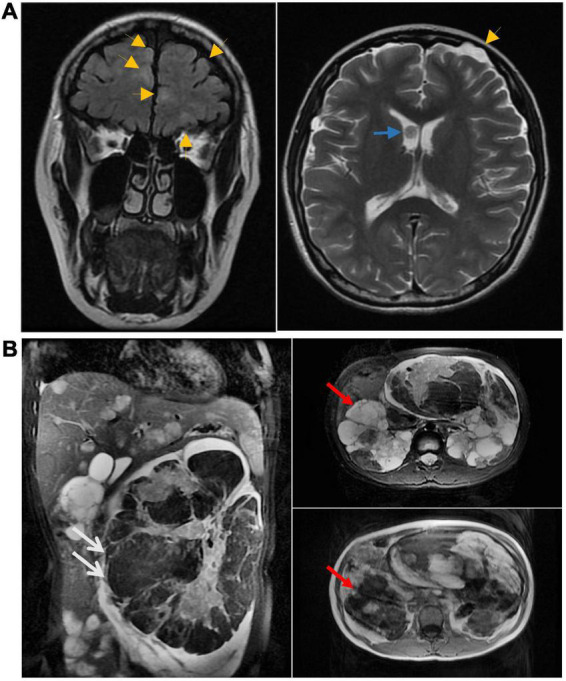
**(A)** Head MRI at 13 weeks of pregnancy showing nodules in the anterior horn of the ventricle (blue arrow). The MRI also showed multiple cortical tubercles (yellow arrow). Multiple lesions were also seen in the subcortical and periventricular areas. **(B)** At 16 weeks of pregnancy, multiple cysts in the liver and kidney and multiple angiomyolipoma were found upon MRI of the upper abdomen. White arrows point to renal vascular smooth muscle lipoma, red arrows point to renal cysts.

At 13 weeks of pregnancy, MRI of the head revealed a nodule in the anterior horn of the ventricle, multiple dysplasia dislocation-like nodules in the cortex and subcortex of both cerebral hemispheres, and subependymal giant cell astrocytoma (Figure 3A). At 16 weeks of pregnancy, multiple cysts in the liver and kidney and multiple angiomyolipoma were found upon MRI of the upper abdomen. A large angiomyolipoma in the left abdominal cavity with a maximum cross-sectional area of 75.4 × 180.4 mm (Figure 3B) was noted. At 30 weeks of pregnancy, another MRI revealed an increased angiomyolipoma with a maximum cross-sectional area of 96 × 182 mm.

## 6. Impression

The patient had bilateral multiple renal cysts, vascular smooth muscle lipoma, and subependymal giant cell astrocytoma (Figures 2, 3). To further clarify the diagnosis, the patient and her parents agreed to undergo whole exome sequencing and multiple ligation-dependent probe amplification (MLPA). To exclude genetic defects in the fetus, the patient also underwent prenatal fetal genetic testing after informed consent.

## 7. Results

**FIGURE 4:**
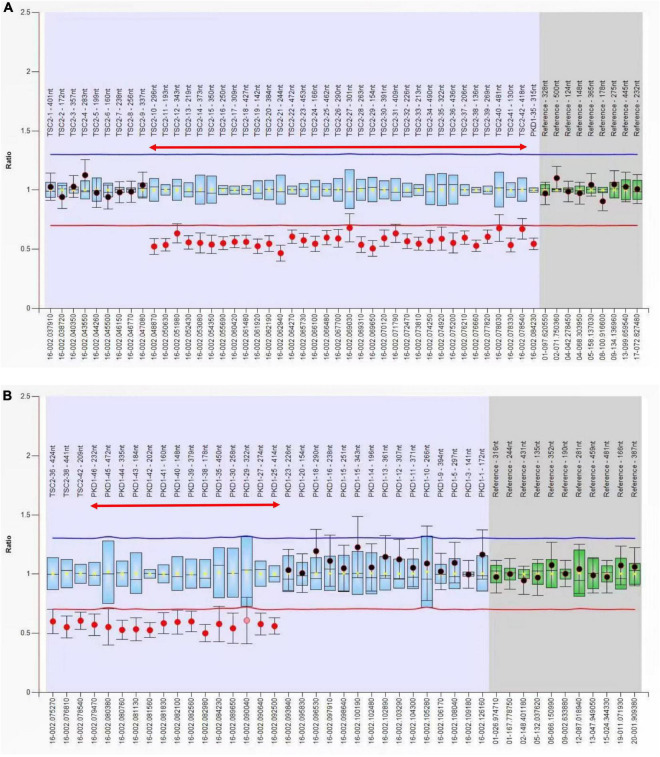
Multiple ligation-dependent probe amplification (MLPA) analysis of the *TSC2* and *PKD1* genes. **(A)** Exon 10–42 deletion of the *TSC2* gene (red two-way arrow). **(B)** Exon 24–46 deletion of the *PKD1* gene (red two-way arrow). The presence of TSC2/PKD1 contiguous gene deletions was confirmed by MPLA molecular studies on the patient.

Whole exome sequencing of the patient’s parents did not reveal pathogenic or potentially pathogenic variants. Whole exome sequencing of the patient revealed a 37.14-kB deletion in the p13.3 region of chromosome 16, including a large heterozygous deletion of *TSC2* exons 11–42 and *PKD1* exons 33–46. No detectable deletions or mutations were found in the exons of the *TSC2* and *PKD1* genes of the fetus. The MLPA molecular study confirmed the presence of deletion of *TSC2* gene exons 10–42 and PKD1 gene exons 24–46 in the maternal samples and confirmed the presence of consecutive deletions in the *TSC2/PKD1* gene (Figure 4). There were no abnormalities in the fetal samples.

## 8. Discussion

Polycystic kidney disease with tuberous sclerosis is a rare, contiguous genomic disease. To date, no more than 90 cases have been identified worldwide; moreover, to our knowledge, no reports of PKDTS in pregnant women have yet been reported. Herein, we have reported the first case of PKDTS in pregnancy presenting with hypomelanotic macules, shagreen patch, subependymal giant cell astrocytoma, angiomyolipomas, bilateral multiple renal cysts, and multiple hepatic cysts. The patient did not present with neuropsychiatric disorders such as epilepsy, and had no abnormalities upon neurological examination. The *PKD1* gene is located 60 bp downstream of *TSC2* in a tail-to-tail orientation. Approximately 2–3% patients with large *TSC2* genomic deletions have a collateral gene deletion, namely the TSC2/PKD1 contiguous gene syndrome (7).

Tuberous sclerosis complex is caused by inactivating mutations in the *TSC1* and *TSC2* genes, which encode the hamartin and tuberin proteins. *TSC1* and *TSC2* are located at positions 9q34 and 16p13.3, respectively (4). These two proteins are widely expressed in normal tissues and combine to form a complex that negatively regulates the mechanistic target of rapamycin (mTOR) complex 1 (mTORC1). mTORC1 activity is important in healthy cells for supporting cell proliferation and differentiation. Tuberin plays a role in the cell cycle (8). mTORC1 plays a role in regulating the bioenergetic and metabolic requirements of cell proliferation and differentiation (4, 9). mTORC1 regulates mTOR-S6K and GTPase activating proteins. mTORC1 plays a central role in the regulation of cell growth and division. When mutations in the *TSC1* and/or *TSC2* genes result in deletion of the complex, they cause aberrant activation of mTORC1, which in turn promotes lipid, nucleotide, and protein synthesis and inhibits cellular autophagy (10–12). Genetic test results can be used as an independent diagnostic criterion for TSC, and approximately 70% TSC patients with mutations in the *TSC2* gene can be identified by genetic testing (13). The failure of routine genetic testing to identify pathogenic variants in the *TSC1* or *TSC2* genes does not exclude the diagnosis of TSC, and patients require long-term clinical monitoring and management for a definitive diagnosis. Patients with TSC can be diagnosed according to clinical and genetic diagnostic criteria (14). It is considered a definite diagnosis if one or two major and two minor features are present, and as suspected diagnosis, if one major or more than two minor features are present (13, 14). Our patient presented with five major features, namely hypomelanotic macules, shagreen patch, subependymal giant cell astrocytoma, multiple cortical tubercles, and angiomyolipomas, and two minor features, namely multiple renal cysts and non-renal hamartomas. Therefore, this patient was definitively diagnosed with TSC by clinical criteria and whole exome sequencing. Imaging by ultrasound and MRI and mutation analysis by prenatal genetic testing may be effective in diagnosing fetuses with TSC and is of high clinical value for early therapeutic intervention in fetuses, particularly in families wherein the child or parents are known to carry the mutation (3).

The most common cause of ADPKD is mutations in the *PKD1* and *PKD2* genes encoding polycystin 1 (PC1) and polycystin 2 (PC2), respectively. *PKD1* and *PKD2* are located at positions 16p13.3 and 4q21, respectively. *PKD1* mutations are responsible for approximately 80% of ADPKD (5). *PKD1* and *PKD2* genes encode for PC1 and PC2 that are localized in the primary cilia. They transmit information from the external environment into the cell. When the level of functional PC1 or PC2 falls below a critical threshold, the likelihood of cyst formation is greatly increased (15). Inhibition of cyst growth and maintenance of renal function are critical in the current treatment of patients with ADPKD. As the disease progresses, the patient’s renal function may decline progressively, requiring enhanced clinical monitoring and management, and even renal replacement therapy if necessary. Ultrasound is now the preferred radiological diagnostic method for ADPKD. MRI and CT are also effective in detecting polycystic kidneys in patients, with greater sensitivity and specificity. Our patient had multiple renal cysts on both ultrasound and MRI with a tendency to increase in size (Figure 2). ADPKD is usually inherited, but new mutations without a family history occur in approximately 10% of cases. A more comprehensive clinical examination for cystic kidney disease is required in patients with a negative family history. In patients aged 16–40 years, the corresponding number of diagnostic cysts detected by MRI is defined as more than 10 to be diagnosed as ADPKD (16). The patient’s diagnosis of PKD was confirmed by ultrasound, MRI and whole exome sequencing.

The *PKD1* and *TSC2* genes are both located on chromosome 16p13.3, and our patient was diagnosed with PKDTS after genetic testing revealed a contiguous gene fragment deletion at this locus. The renal manifestations of PKDTS are dominated by cysts, and the early onset, size, and number of cysts are the main features (17). There are currently no definitive treatment options for PKDTS for clinical use. Patients’ kidneys often enter end-stage renal disease very early, mostly in childhood and adolescence and, to a lesser extent, in early adulthood; hence, many patients are already in end-stage renal disease at diagnosis and require renal replacement therapy (5, 18). Renal transplantation is likely to remain the preferred renal replacement therapy option at present, but reports of renal transplantation in patients with PKDTS are scarce, so immunosuppressive therapy may be one of the main treatment options in the future. The use of mTORC1 inhibitors such as sirolimus (also known as rapamycin) has become one of the important therapeutic measures for patients with TSC, but more clinical studies are needed to better understand the efficacy of these drugs in patients with PKDTS during pregnancy and determine whether they have any adverse effects on the fetus. From this case report, we found that the burden on the kidneys of patients with PKDTS during pregnancy may be further increased and renal function further decreased, allowing patients to rapidly progress to end-stage renal disease. Therefore, nephrotoxic drugs should be avoided in patients with PKDTS, especially if they are pregnant. Notably, total kidney volume is considered in PKD not only as a marker of disease progression but also as a test of treatment efficacy (19).

In this case, the large abdominal mass detected by ultrasound was diagnosed as RAML with blood supply from the left renal artery (Figure 2). RAML is a rare benign renal tumor. The tumor tissue in RAML comprises blood vessels, smooth muscle, and fat and is one of the main symptoms commonly seen in patients with typical TSC (4). Patients with RAML during pregnancy may experience fatal complications such as rupture and hypovolemic shock due to bleeding. It is now generally accepted that the main factor for RAML rupture is the elevated expression of estrogen and progesterone receptors in the tumor (20). Moreover, increased maternal circulation and abdominal pressure during pregnancy play an important role in the rupture of RAML (21). In conclusion, RAML in pregnant patients tend to grow and rupture as the pregnancy advances. By reviewing clinical guidelines and relevant reports, the treatment of pregnant patients with RAML should be individualized according to the patient’s hemodynamic status and fetal maturity. Patients with RAML can be treated conservatively when they are hemodynamically stable and should be managed with intensive clinical monitoring and follow-up. When acute rupture or hemodynamic instability is present, either emergency surgery or selective arterial embolization (SAE) can be an option, but in cases of acute ruptured hemorrhage, SAE is often chosen (20, 22).

In summary, patients with PKDTS often have symptoms of both TSC and ADPKD, and the symptoms are typically more severe than those of TSC or ADPKD alone. In pregnant patients with PKDTS, the focus should be on the patient’s renal manifestations, including multiple renal cysts and RAML. Enhanced clinical monitoring and follow-up with an individualized treatment plan is essential to improve the patient’s renal function and obtain the desired pregnancy outcome. We have found through clinical monitoring and follow-up that patients with PKDTS during pregnancy have enlarged renal cysts and RAML. To date, no progressive deterioration in renal function has occurred during conservative treatment of pregnant women with PKDTS. Confirmation of whether PKDTS patients have accelerated progression to end-stage renal disease during pregnancy and whether fatal complications such as ruptured RAML bleeding may ultimately lead to adverse pregnancy outcomes requires more clinical data. Our patient’s hemodynamic status and fetal maturity were fully assessed and a conservative treatment plan was decided upon after prenatal genetic testing ruled out fetal genetic defects to achieve the best possible outcome for both mother and fetus. We monitored the patient until 31 weeks of gestation. The fetal growth and development were good, and the patient’s vital signs were stable. No abnormal results in genetic testing of fetal samples.

## 9. Conclusion

There are currently no diagnostic criteria and clinical guidelines for PKDTS. There is a considerable lack of experience in the management of this disease, which is both a clinical challenge and risk for accurate management of such patients. To the best of our knowledge, this is the first case report to describe the clinical presentation and characteristics of PKDTS in a pregnant patient. We have focused on the main clinical features of patients with PKDTS in pregnancy, particularly the renal manifestations. We found an increasing trend in the size of renal cysts and RAMLs in patients with PKDTS during pregnancy. Through enhanced clinical monitoring of patients and prenatal genetic testing of the fetus, timely and effective clinical intervention for the mother may be achieved, thus obtaining the best possible outcome for both the mother and fetus.

## Data availability statement

The original contributions presented in this study are included in the article/supplementary material, further inquiries can be directed to the corresponding authors.

## Ethics statement

Written informed consent was obtained from the individual(s) for the publication of any potentially identifiable images or data included in this article.

## Author contributions

XH designed this study. SH and YX conducted the data collection and analysis. SH and KX drafted the manuscript which was checked by LZ and XH. All authors contributed to the article and approved the submitted version.
